# The Climate of the United States, and Its Endemic Influences, &c.

**Published:** 1842-07-01

**Authors:** 


### The Climate of the United States, and its Endemic Influences, &c. &c. By *Samuel Forry*, M.D. New York, 1842.

The multitudes of our countrymen who cross the Atlantic in pursuit of commerce, or led by curiosity, render a work of this kind very desirable, especially as no publication of the kind existed previous to this, except perhaps the vague remarks of Volney, more than 40 years ago. This volume is based chiefly on the statistical reports of the army, and embraces a period of 20 years, viz. from 1819 to 1839—the result of our author's labours.

The United States are spread over a vast space of 2,300,000 square miles—stretching from the Gulf of Mexico to the Russian and British possessions on the North—from the Atlantic on the West to the Pacific on the East!! This huge territory is traversed by two great systems of mountains—the more lofty of which (Rocky, Oregon, and Chippewyan) is a prolongation of the Mexican Cordilleras, extending to the Arctic regions—varying from eight to ten thousand feet above the level of the sea. The general face of the country presents every variety of mountain, valley, and table-land, composed of primitive, transition, secondary, and alluvial formations. Two mighty rivers, the Mississippi and St. Lawrence, drain the vast territory, South and North, while numerous subordinate rivers open on the eastern sea-board, forming great estuaries and noble harbours for commercial navies or warlike fleets.

More than half of the volume before us is dedicated to medico-statistical details of the various military posts and stations of the three great divisions of the States—Northern, Middle, and Southern. Into these we cannot possibly enter. But, from the second part, entitled "General Deductions," we shall endeavour to glean some portions of curious or even important information.

### I. Pulmonary Diseases.

"Occupying, as we do, the eastern coast of a continent of the northern hemisphere, the human frame is exposed to the contrasted seasons of the most excessive clime. The extreme north has a climate in which cold predominates, vexed by winds that have passed over interminable snows; the south acknowledges the genial influence of the sun; whilst the middle vibrates alternately to both extremes. The climate of the United States is, in truth, remarkably inconstant and variable, 'passing rapidly,' says Malte-Brun, 'from the frosts of Norway to the scorching heats of Africa, and from the humidity of Holland to the drought of Castile.' So sudden are the vicissitudes of weather in the middle States, that it may be truly said, we often 'lie down in July and rise in December.'" 229.

From this description we should not augur well for the pulmonary apparatus.

From a table at page 231, we find that the average number of catarrhs in one thousand men, was 287 in the year; but varying greatly according to the quarter of the year, and the division of the territory. Thus in the first or spring quarter, it was 98—in the second quarter 56—in the third, 42—and in the 4th, 88. In the northern and southern divisions of America, the difference was still more striking. In the northern parts of America, catarrhs averaged 552 in the thousand—in the southern, only 143, in the same number. The different quarters of the year, in the different States, presented great numerical differences also. The author strongly insists on the advantages of moving to the southern States, in cases of bronchial affections, the sequences of catarrhal attacks.

"The investigation of catarrhal diseases in reference to the agency of climate, and especially the seasons, is thus concluded; and the results, it is conceived, demonstrate conclusively the advantage of a winter residence in the peninsula of Florida in cases of *chronic bronchitis.*" 237.

### PLEURITIS AND PNEUMONIA.

In the three divisions of the United States, the annual average of the above complaints was 53 in the 1000 mean strength; but in some States, as in the south-western stations, it was as high as 92 in the thousand; while in other places, as the peninsula of East Florida, it only amounted to 39 in the same number of men.

### PHTHISIS.

The total annual average of this disease was nine in the thousand men; but varying in different stations from five to thirteen. The highest ratio was from Delaware Bay to Savannah; and the lowest was in posts remote from the ocean and inland seas. The peninsula of East Florida averaged nine in the thousand.

### INTERMITTENTS.

This malarious scourge plays an important part in the statistics of sickness in America.

The annual average of all the States is 368 in a thousand men! There is a variation in different states and stations of 36 to 747. From Delaware Bay to Savannah the high rate is shewn. The coast of New England presents the small number of 36. The total average of remittent fevers is comparatively small, viz. 101—varying from 24 to 196. Typhus fever is apparently rare, averaging only $3\frac{1}{2}$ in the thousand. The average mortality from all fevers was $4\frac{1}{2}$ in thousand.

### DIARRHŒA AND DYSENTERY.

These are very frequent complaints, averaging annually 405 in a thousand troops. The autumnal quarter shews thrice the number of any quarter.

We are unable to go farther into this valuable volume, as it entirely defies analysis. We shall conclude with the following short extract relative to the fatal and wide-spread epidemic which frightened the two worlds in the form of cholera.

"The contagious nature of this epidemic is rendered still more questionable from the fact, confirmed with little exception, by the whole current of medical testimony in Europe, Asia, and America, that neither physicians nor those in constant attendance exhibited any peculiar liability to it. Medical officers have slept in their hospitals; nurses, to quiet timid females, have shared their beds during the night; the bed-clothes of patients who have died have been immediately used; and yet no bad consequences have followed. At Warsaw, Dr. Foy inhaled the breath, tasted the dejections, and inoculated himself with the blood of patients, without contracting the disease. There remains, however, another fact which seems the *experimentum crucis*, viz. that thousands of persons left infected districts, and died of the disease in various places, without communicating it to the surrounding inhabitants." 321.

